# Novel Homozygous FA2H Variant Causing the Full Spectrum of Fatty Acid Hydroxylase-Associated Neurodegeneration (SPG35)

**DOI:** 10.3390/genes15010014

**Published:** 2023-12-20

**Authors:** Alexander German, Jelena Jukic, Andreas Laner, Philipp Arnold, Eileen Socher, Angelika Mennecke, Manuel A. Schmidt, Jürgen Winkler, Angela Abicht, Martin Regensburger

**Affiliations:** 1 Department of Molecular Neurology, Friedrich-Alexander-Universität Erlangen-Nürnberg (FAU), 91054 Erlangen, Germany; 2 MGZ—Medizinisch Genetisches Zentrum, 80335 Munich, Germany; 3 Institute of Functional and Clinical Anatomy, Friedrich-Alexander-Universität Erlangen-Nürnberg (FAU), 91054 Erlangen, Germany; 4 Institute of Neuroradiology, Friedrich-Alexander-Universität Erlangen-Nürnberg (FAU), 91054 Erlangen, Germany; 5 Center for Rare Diseases (ZSEER), University Hospital Erlangen, 91054 Erlangen, Germany

**Keywords:** hereditary spastic paraplegia, SPG35, FA2H, FAHN, genetic variant modeling

## Abstract

Fatty acid hydroxylase-associated neurodegeneration (FAHN/SPG35) is caused by pathogenic variants in *FA2H* and has been linked to a continuum of specific motor and non-motor neurological symptoms, leading to progressive disability. As an ultra-rare disease, its mutational spectrum has not been fully elucidated. Here, we present the prototypical workup of a novel *FA2H* variant, including clinical and in silico validation. An 18-year-old male patient presented with a history of childhood-onset progressive cognitive impairment, as well as progressive gait disturbance and lower extremity muscle cramps from the age of 15. Additional symptoms included exotropia, dystonia, and limb ataxia. Trio exome sequencing revealed a novel homozygous c.75C>G (p.Cys25Trp) missense variant in the *FA2H* gene, which was located in the cytochrome b5 heme-binding domain. Evolutionary conservation, prediction models, and structural protein modeling indicated a pathogenic loss of function. Brain imaging showed characteristic features, thus fulfilling the complete multisystem neurodegenerative phenotype of FAHN/SPG35. In summary, we here present a novel *FA2H* variant and provide prototypical clinical findings and structural analyses underpinning its pathogenicity.

## 1. Introduction

Hereditary spastic paraplegias (HSPs) are a group of genetically and clinically diverse neurological disorders, which are characterized by progressive lower extremity weakness and spasticity due to a length-dependent axonal degeneration of the corticospinal tracts [1]. The spastic paraplegia 35 subtype (SPG35, MIM 612319) shows a “complex” HSP phenotype, i.e., additional symptoms including upper limb spasticity, truncal instability, dysarthria, dysphagia, cerebellar ataxia, and progressive cognitive deficits [2]. SPG35 is caused by biallelic pathogenic variants in the *FA2H* gene, which encodes fatty acid 2-hydroxylase (FA2H), an enzyme that functions in α-hydroxylation of free fatty acids in the endoplasmic reticulum of oligodendrocytes [3]. These fatty acids are mainly incorporated into galactosylceramide and -sulfatide [3]. Approximately 25% of the outer leaflet lipids in myelin are 2-hydroxylated [4,5], with a possible role of increasing membrane fluidity [6]. Patients with SPG35 exhibit a narrow brain imaging phenotype previously described by the ‘WHAT’ acronym: stationary White matter changes, Hypointensity of the globus pallidus, pontocerebellar Atrophy, and Thin corpus callosum [2]. In a previous case series, 85% of patients exhibited three or more WHAT features [2]. In all but two published cases, gait disorder manifested in early childhood, and the use of a wheelchair was required after a median of 7 years [2]. Furthermore, 50% of patients developed exotropia, and unusually bristle hair was noted in several patients. Scanning electron microscopy of hairs revealed longitudinal grooves in all four out of four analyzed patients and adhesive plaques in three out of four patients. The aim of our study was to validate a novel *FA2H* variant in an early-onset HSP patient by precise clinical phenotyping as well as in silico analyses. Our data demonstrate that the c.75C>G *FA2H* variant is associated with the prototypical phenotypic presentation of SPG35 and that this variant is predicted to lead to an enzymatic dysfunction.

## 2. Materials and Methods

The presented subject gave informed consent for analyses before participation in the study. The study was conducted in accordance with the Declaration of Helsinki and was approved by the Ethics Committee of the Friedrich-Alexander-Universität Erlangen-Nürnberg (no. 17-259_3-B).

### 2.1. Genetics

#### 2.1.1. High Throughput Sequencing and Bioinformatics Pipeline

Next-generation sequencing analysis (NGS) was carried out on an Illumina NovaSeq 6000 system (Illumina, San Diego, CA, USA) as 150 bp paired-end sequencing runs using v2.0 SBS chemistry. Sequencing reads were aligned to the human reference genome (GRCh37/hg19) using BWA (v0.7. 13-r1126) with standard parameters. SNV, CNV, and INDEL calling on the genes were conducted using the varvis software platform (varvis™; Limbus Medical Technologies GmbH, Rostock, Germany) with subsequent coverage and quality-dependent filter steps.

#### 2.1.2. Nomenclature, Interpretation, and Classification of Genetic Variants

The nomenclature guidelines of the Human Genome Variation Society (HGVS) were used to describe DNA sequence variants [7]. Population databases were used to assess the allele frequencies of the variants: Database of all known Single Nucleotide Polymorphisms (dbSNP), Exome Aggregation Consortium (ExAC), and Genome Aggregation Database (gnomAD).

### 2.2. Structural Modeling of the Cys25Trp Variant

A structural model created with AlphaFold v2 [8,9] was taken from UniProt (Entry: Q7L5A8) for the wild type because there is no experimentally solved protein structure of FA2H so far. The human FA2H consists of two distinct domains: the N-terminal cytochrome b5-like domain containing the heme group (residues 15–85) and the C-terminal sphingolipid fatty acid hydroxylase domain (residues 124–366) [10]. In order to identify the most similar experimentally determined structures for each of the two domains, a protein blast with NCBI was performed. The structure of type B cytochrome b5 (PDB ID code: 3NER, [11]) was found as the most similar human and experimentally determined structure for the N-terminal domain, and the position of its heme group was used as reference after structure superposition. For the C-terminal hydroxylase domain, NCBI protein blast identified the hydroxylase domain of scs7p from Saccharomyces cerevisiae S288C (PDB ID code: 4ZR0, [12]) to be the most similar experimentally determined structure. For illustration of how FA2H is oriented in the endoplasmic reticulum membrane, FA2H was positioned in a model membrane according to the Orientations of Proteins in Membranes (OPM) database (https://opm.phar.umich.edu/, accessed on 10 October 2023) entry to PDB ID code: 4ZR0. AlphaFold protein structure prediction for the mutant FA2H (Cys25Trp) was performed using ChimeraX [13,14] and ColabFold [15]. The 2D ligand interaction diagrams for the protein-heme interactions were generated using the academic version of Maestro 13.6 (Schrödinger Release 2023-2: Maestro, Schrödinger, LLC, New York, NY, USA, 2023). All protein structure images were generated with UCSF Chimera 1.16 [13].

### 2.3. Scanning Electron Microscopy of Hair Shafts

Scanning electron microscopy (SEM) was performed as described before [16,17]. Unlike for hydrated samples such as cells, we did not dry the samples at a critical point but sputtered them directly with gold after glue fixation on aluminum specimen mounts. Samples were transferred to a JEOL JSM-IT300 scanning electron microscope, and images were acquired at 5 kV acceleration voltage and 400× magnification.

## 3. Results

### 3.1. Case Report

**Figure 1:**
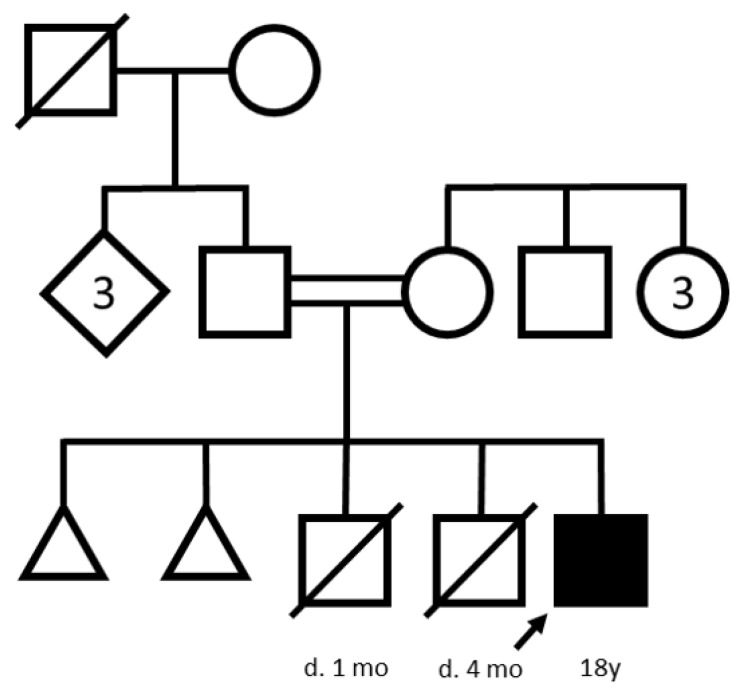
Pedigree of the index patient (arrow) according to [18].

An 18-year-old cis male patient of Turkish descent presented with a history of cognitive impairment and progressive gait disturbance. The patient’s family history was notable for the consanguinity of his parents, who are cousins. The mother has four healthy siblings. She reported two spontaneous abortions and the premature death of two male children at 1 at 4 months, respectively, for unknown reasons. The father reported three healthy siblings, but a gait disorder in his deceased father (i.e., the index patient’s grandfather) starting at age 40. The pedigree according to [18] is shown in Figure 1.

The patient’s cognitive and physical development was described as unremarkable during early childhood. Nevertheless, learning difficulties manifested during primary school with unaffected physical abilities, which were described as athletic. At the age of 15, he developed a slowly progressive gait disorder and calf cramps. By age 18, he required not only increasing help with reading and writing but also for orientation due to cognitive decline. His walking distance was limited to approximately 100 m, after which he needed to rest due to an increasing risk of falling. He had experienced several falls and reported muscle pain in the thigh and forearm muscles. His mood remained stable, al-though he occasionally expressed frustration due to increasing disability and social isolation. At age 18, the patient presented as being cognitively slow, but fully oriented. Emotional affect was blunted, with a low state of mood. In terms of ocular exam, he exhibited horizontal exotropia and occasional double vision. Facial tone was slightly reduced, and speech was mildly dysarthric. Motor signs included spastic paraparesis, overall bradykinesia, and intermittent dystonia of the fingers and arms. Tendon reflexes were brisk in the arms and clonic in the legs; there were positive Babinski and palmomental reflexes bilaterally. In addition, coordination tests were ataxic. The walking pattern was spastic-ataxic with a widened base and signs of central hip muscle weakness, leading to an increased risk of falling, especially during running. The patient’s hair had a bristle-like appearance. Electromyography examinations were unremarkable.

The patient’s treatment plan included physiotherapy including intensive therapy in a neurological rehabilitation clinic and the introduction of occupational therapy and speech therapy. Pain reduction was partly achieved by gabapentin 1200 mg/d, while no additional benefit was reported at 1600 mg/d.

**Figure 2:**
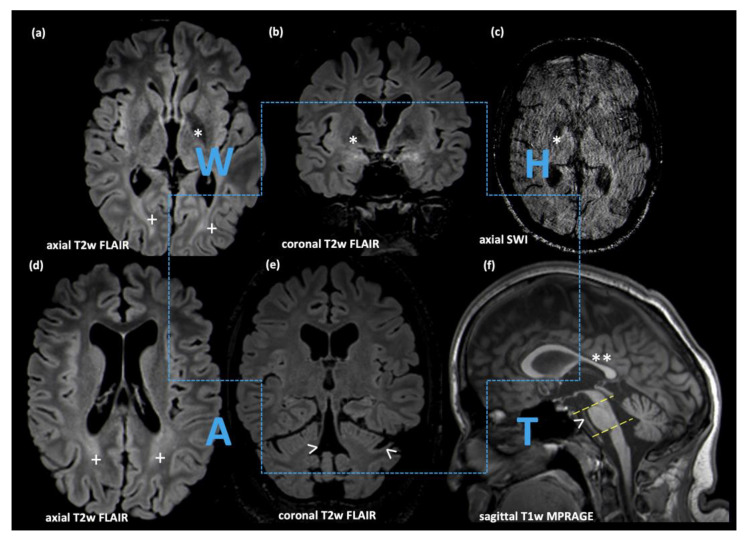
MRI Findings. “WHAT” tetrad: spotty FLAIR-hyperintensities affecting the parietal white matter (“W”; cross symbol in (**a**,**d**)), hypointensity of the globus pallidus in T2w FLAIR/SWI (“H”; asterisk in (**a**–**c**)), pontocerebellar atrophy with decreased midbrain but not pons midsagittal area (“A”; open arrows in (**e**,**f**) (the pontomesencephalic junction is defined by the yellow dotted line between the superior pontine notch and the inferior border of the quadrigeminal plate; the pontomedullary junction is defined by a line parallel to this line at the level of the inferior pontine notch)) and thinning of the corpus callosum, predominantly affecting the body (“T”; double-asterisk in (**f**)).

Magnetic resonance imaging (MRI) of the brain revealed the complete tetrad of “WHAT” (Figure 2), i.e., biparietal, spotty FLAIR-hyperintensities (“W”), hypointensity of the globus pallidus in T2w FLAIR and susceptibility-weighted imaging (“H”), pontocerebellar atrophy with widening of the fourth ventricle and decreased midbrain (361 mm^2^) but not pons midsagittal area (129 mm^2^) (“A”) and thinning of the corpus callosum (“T”). AI-supported volumetry revealed atrophy of the thalamus (6.1 mL), midbrain (6.3 mL), and pons (8.9 mL), each with more than four standard deviations. Of note, the magnetic susceptibility of the globus pallidus was markedly reduced at 62 ppb, as measured by Quantitative Susceptibility Mapping (QSM, [19]). Imaging findings were stable at a 3.5-year follow-up. Spine MRI was unremarkable at age 15.

### 3.2. Molecular Genetic Analysis

**Figure 3:**
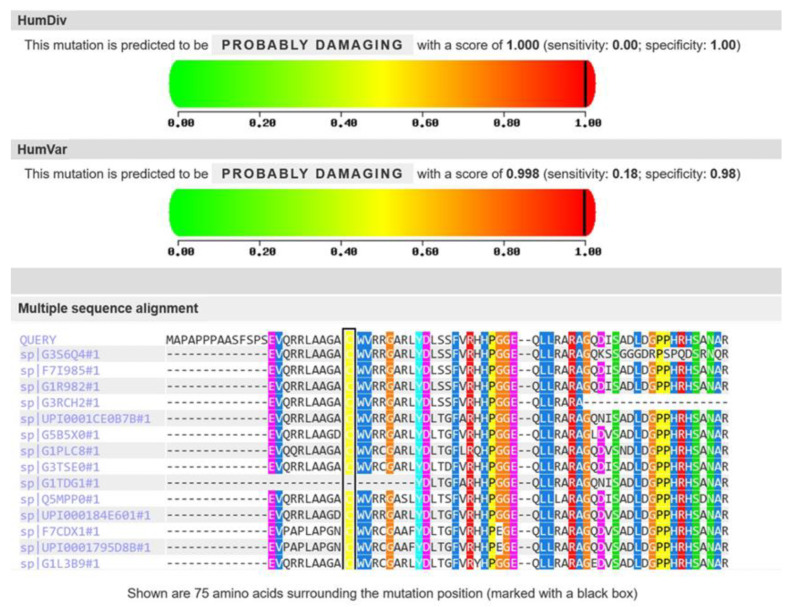
PolyPhen-2 shows strong preservation of cysteine at position 25 in FA2H and predicts “probably damaging” for the exchange with tryptophan.

Genetic trio-exome analysis including the patient and both parents revealed a homozygous c.75C>G missense variant in the *FA2H* gene, with the same monoallelic alteration present in both consanguineous parents. The cysteine is highly preserved across species, with predicted pathogenicity at the amino acid level for the variant according to PolyPhen-2 [20,21] (Figure 3).

The bioinformatic meta-predictor MetaSVM [22] suggested a damaging effect (MetaSvmRank: Damaging (0.88508); a rank above 0.82 is regarded as damaging). REVEL [23] prediction was 0.737, agreeing with the MetaSVM classification as PP3 [24]. Accordingly, the variant was classified according to the ACMG guidelines with the five-tier classification system [25] as a class 3 (variant of uncertain significance, VUS; PM1, PM2_SUP, PP3). The variant was submitted to LOVD (https://databases.lovd.nl/shared/individuals/00435460, accessed on 10 October 2023) and ClinVar (submission# SCV002578919.1).

### 3.3. Structural Modeling of the Cys25Trp Variant

**Figure 4:**
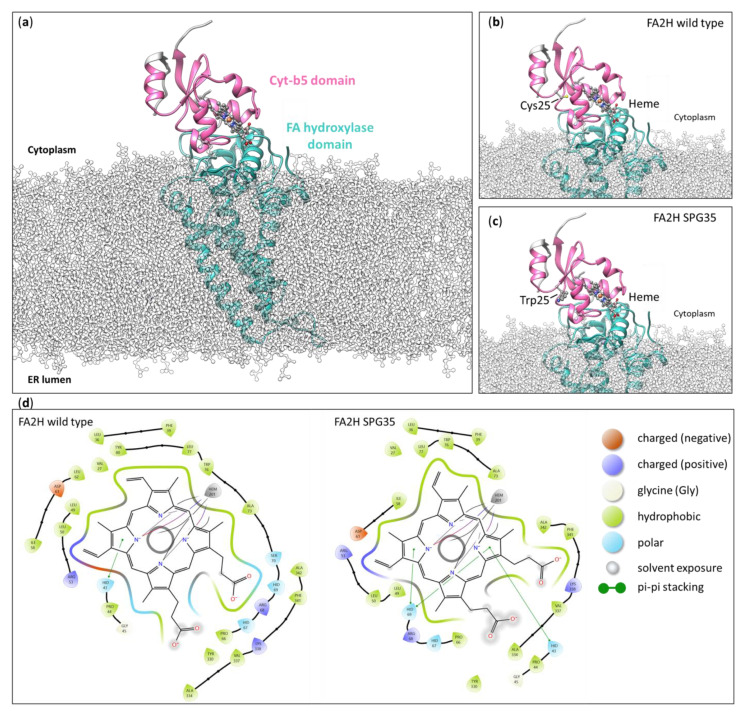
Structural modeling of the Cys25Trp variant. (**a**) Overview of the FA2H structural model (AlphaFold) in a model membrane. While the FA hydroxylase domain inserts into the membrane, the Cyt-b5 domain remains accessible at the cytoplasm. (**b**) Detailed depiction of cysteine 25 (Cys25) in the Cyt-b5 domain in proximity to the iron-binding heme domain (heme). (**c**) Structure of the Cyt-b5 domain with a tryptophan at position 25 (Trp25). (**d**) A 2D representation of the heme group with residues in <5 Å distance. The coordination between both variants (FA2H wild type and SPG35) differs, which might result in inferior enzymatic activity in SPG35 patients.

Protein structures of wild type and mutant FA2H (cysteine 25 to tryptophan: Cys25Trp) were predicted by AlphaFold [8,9]. They were docked into a lipid membrane with the FA hydroxylase domain and leaving the Cyt-b5 domain oriented towards the cytoplasm (Figure 4). Residue 25 resides within the Cyt-b5 domain in proximity to the heme group, which is important for the coordination of the iron ion required for enzymatic activity (Figure 4).

### 3.4. Scanning Electron Microscopy of Hair Shafts

**Figure 5:**
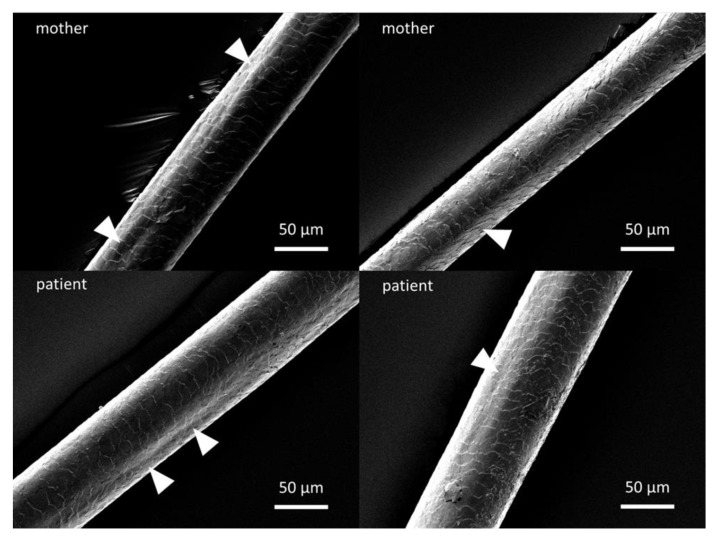
Scanning electron microscopy (SEM) analysis of scalp hair. Comparative SEM analysis of hairs from the patient (**bottom**) and his mother (**top**) revealed a comparable cuticle structure. Both showed groove structures (white arrows) to a comparable extent. Scale bars 50 µm.

Potential groove formations in the patient’s scalp hair as well as in the control hair from his mother were analyzed using scanning electron microscopy. Both patient and control hair samples showed a typical cuticle overlay expected for healthy hair. In accordance with [2], we assessed longitudinal groove formation in hair shafts, comparing patient and control. We identified grooves in both the patient and control hair samples (Figure 5, white arrows), without apparent differences with regard to depth or length, however. Specifically, no adhesive plaques were present.

## 4. Discussion

In the present report, we demonstrate the phenotype of a novel homozygous c.75C>G missense variant in *FA2H*. The presented case shows the full spectrum of characteristic imaging and clinical findings in SPG35 [2]. Differential clinical diagnoses including MPAN (mitochondrial membrane protein-associated neurodegeneration) and CoPAN (COASY protein-associated neurodegeneration) were unlikely due to lack of parkinsonism. SPG11, SPG15, and GAN (Giant axon neuropathy) were unlikely due to the absence of muscular atrophy [2]. Finally, *FA2H* was the only gene prioritized in trio exome sequencing.

Due to the clinical and imaging findings characteristic of SPG35, in combination with protein structure predictions, the c.75C>G variant of the *FA2H* gene is likely to lead to a (partial) loss of function. In SPG35, gait disorder was reported to occur at a median age of 4, with an interquartile range of 3–4.5 [2], whereas our patient developed gait disorder at age 15. In accordance with the present structural model, Cys25Trp FA2H might possess residual catalytic activity. Alternatively, the delayed onset of gait disorder in our patient as compared to typical early onset SPG35 cases might be caused by modifier genes or environmental factors influencing disease progression. A comprehensive overview of published pathogenic variants in the *FA2H* gene is listed in Table 1. The discovery of this novel variant highlights the evolving understanding of the genetic underpinnings of oligodendroglial dysfunction, leukodystropy, and neurodegenerative disorders, suggesting that there may be other yet-to-be-discovered mutations contributing to diverse presentations of SPG35.

As opposed to a previous report [2] and despite the bristle-like hair appearance in the presented patient, SEM did not reveal a hair shaft anomaly. We conclude that such SEM features of hair may not be consistent sensitive markers of SPG35.

## 5. Conclusions

Our data corroborate the pathogenicity of the novel c.75C>G variant in *FA2H* and contribute to the understanding of *FA2H*-associated HSP, underscoring the need for further research into effective treatments for this rare condition.

## Figures and Tables

**Table 1:** Overview of reports of FA2H variants with SPG35 phenotypes.

Article	FA2H Variant
[26] Dick	c.703C>T, c.159_176del18
[2] Rattay	c.21delC, c.160_169del GCGGGCCAGG, c.205C>T, c.232G>A, c.262G>T, c.443C>T, c.503_506del TCTG, c.704G>A, c.859T>C, c.908G>T, c.956A>G
[27] Soehn	c.131C>A, c.133G>T, c.527G>A, c.785A>C
[28] Kruer	c.460C>T, c.510_511delCA
[29] Pensato	c.620C>T
[30] Rupps	c.209C>T, c.968C>T
[31] Edvardson	c.103G>T, c.786+1G>A
[10] Mari	c.193C>T, c.805c>T, c.1055C>T, c.1501A>G, [c.340_363del24][c.363+1_8del8]
[32] Hashemi	c.131delC
[33] Incecik	c.130C>T
[34] Bektaş	c.160_169dup
[35] Liao	c.388C>T, c.506+6C>G, c.230T>G
[36] Zaki	c.265C>T
[37] Tonelli	c.509A>G
[38] Aguirre-Rodriguez	C565C>T
[39] Pierson	c.707C>T
[40] Cao	c.968C>A; c.976G>A; c.688G>A
This report	c.75C>G

## Data Availability

The data presented in this study are available on request from the corresponding author. The data are not publicly available due to identifying information.
